# Infrared thermography videos of the elastocaloric effect for shape memory alloys NiTi and Ni_2_FeGa

**DOI:** 10.1016/j.dib.2015.07.011

**Published:** 2015-07-30

**Authors:** Garrett J. Pataky, Elif Ertekin, Huseyin Sehitoglu

**Affiliations:** Department of Mechanical Science and Engineering, University of Illinois at Urbana-Champaign, 1206 W. Green Street, Urbana, IL 61801, United States

## Abstract

Infrared thermogrpahy was utilized to record the temperature change during tensile loading cycles of two shape memory alloy single crystals with pseudoelastic behavior. During unloading, a giant temperature drop was measured in the gage section due to the elastocaloric effect. This data article provides a video of a [001] oriented Ni_2_FeGa single crystal, including the corresponding stress–strain curve, shows the temperature drop over one cycle. The second video of a [148] oriented NiTi single crystal depicts the repeatability of the elastocaloric effect by showing two consecutive cycles. The videos are supplied in this paper. For further analysis and enhanced discussion of large temperature change in shape memory alloys, see Pataky et al. [1]

Specifications table

**Table:** 

Subject area	Materials
More specific subject area	Shape memory alloys
Type of data	Videos
How data was acquired	Infrared thermography, extensometer
Data format	Analyzed
Experimental factors	*The samples were heat treated as indicated in* Ref. [1]*. The back of the samples were lightly painted black for uniform emissivity.*
Experimental features	An infrared camera captured the temperature changes during a tensile cycle, while a 5 mm gauge length extensometer measured the strain in the gauge section.
Data source location	N/A
Data accessibility	Data with article

**Value of the data**

• The videos included in this brief capture the large temperature change in shape memory alloys due to the elastocaloric effect, important for further development of solid state refrigeration.
• The two consecutive cycles of the NiTi specimen show symmetry between the first and second cycle proving stability of the temperature change.
• Both shape memory alloys utilized do not include rare earth metals and have large temperature changes. This is important for affordable solid state refrigeration.

**Data**

Two videos provided here capture the large temperature change in shape memory alloys, NiTi and Ni_2_FeGa, due to the elastocaloric effect.

## 1 Materials and methods

Two shape memory alloys were utilized: NiTi and Ni_2_FeGa. The NiTi material was nickel-rich at 50.375 at% and the Ni_2_FeGa material had a composition of Ni_54_Fe_19_Ga_27_ (at%). Single crystal specimens for each material were grown using the Bridgman technique. The NiTi material was solutionized for 24 h at 920 °C, then heat treated at 550 °C for 1.5 h. Using differential scanning calorimetry (DSC), the characteristic temperatures were found to be *A*_f_=0 °C, *A*_s_=−15 °C, *M*_s_=−55 °C, and *M*_f_=−75 °C. The material undergoes a B2–B19׳ transformation. The Ni_2_FeGa material was unaged, and a DSC analysis was used to find characteristic temperatures of *A*_f_=22 °C, *A*_*s*_=14 °C, M_s_=6 °C, and *M*_f_=−3 °C. Upon stressing, the material undergoes a transformation of L1_2_→10 M→14 M→L1_0_.

Tensile experiments were performed on each specimen and the temperature change due to the phase transformations were captured using infrared thermography. An extensometer with a 5 mm gauge length was used to record the stress–strain behavior. During loading, the austenite transformed to stress-induced martensite causing an increase in the temperature of the material. During unloading, the reverse martensitic transformation occurred with a corresponding large temperature drop.

In order to capture the full temperature drop, the unloading strain rate was 10^−2^ s^−1^, approaching adiabatic conditions. The Ni_2_FeGa specimen was strained to approximately 10%, shown in Video 1, while the NiTi specimen was strained to 4.25% for both cycles, shown in Video 2. A temperature change of approximately 8 °C was measured during unloading in the [001] Ni_2_FeGa specimen, as seen in Video 1, and 14.6 °C in the [148] NiTi specimen, as seen in Video 2.

## Figures and Tables

**Video 1:**
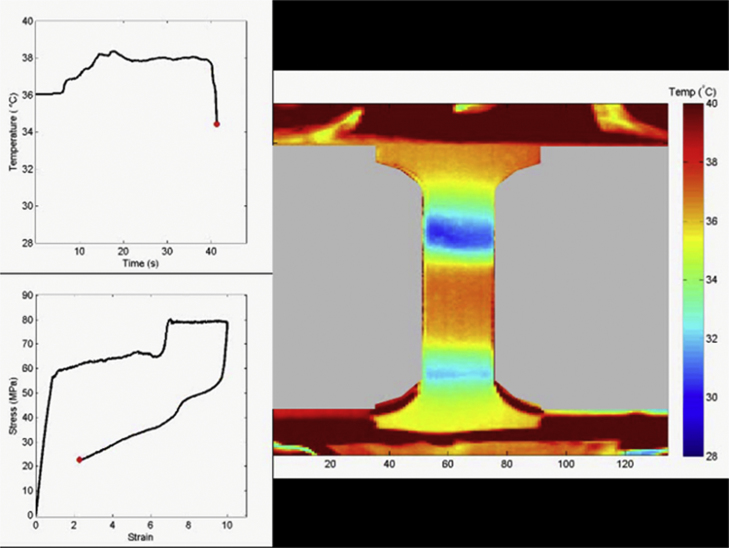
showing the tensile cycle of the [001] oriented Ni_2_FeGa specimen. The upper right plot shows temperature versus time, and the plot below shows the stress–strain plot. A video clip is available online. Supplementary material related to this article can be found online at doi:10.1016/j.dib.2015.07.011.

**Video 2:**
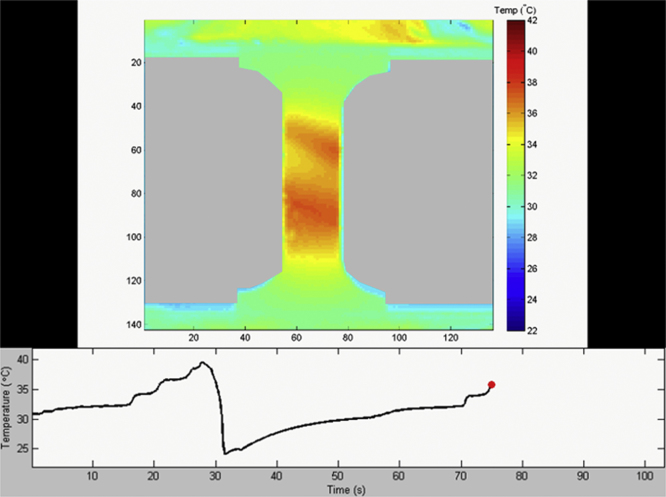
showing two consecutive tensile cycles of the [148] oriented NiTi specimen. The temperature evolution versus time plot is shown at the bottom. A video clip is available online. Supplementary material related to this article can be found online at doi:10.1016/j.dib.2015.07.011.
